# Hydrogel-Transformable Antioxidant Poly-γ-Glutamic Acid/Polyethyleneimine Hemostatic Powder for Efficient Wound Hemostasis

**DOI:** 10.3390/gels10010068

**Published:** 2024-01-17

**Authors:** Xiang Li, Wenli Han, Gao He, Jiahao Yang, Jing Li, Hongxia Ma, Shige Wang

**Affiliations:** 1 School of Public Health, Center for Global Health, Nanjing Medical University, Nanjing 211100, China; 2 School of Materials and Chemistry, University of Shanghai for Science and Technology, No. 516 Jungong Road, Shanghai 200093, China

**Keywords:** hemostatic powder, hydrogel, γ-polyglutamic acid, polyethyleneimine, wound hemostasis

## Abstract

Hemostatic powder, which can absorb large amounts of water and tends to produce repeated hydration with tissue, has been clinically proven as an ideal engineering material for treating wounds and tissues. We herein designed a polypeptide-based hemostatic powder. A water-soluble polypeptide, γ-polyglutamic acid (γ-PGA), was mixed with the polyethyleneimine (PEI), N-hydroxysuccinimide, and 1-(3-dimethylaminopropyl)-3-ethylcarbodiimide. The solution of these polymers was lyophilized to harvest the γ-PGA/PEI powder (PP hemostatic powder). When deposited on a bleeding wound, the PP hemostatic powder can quickly absorb a large amount of blood and interstitial fluid, concentrate coagulation factors, coagulate blood cells, and eventually form a stable mechanical hydrogel. The wound bleeding time of the PP hemostatic powder group was 1.8 ± 0.4 min, significantly lower than that of the commercial chitosan hemostatic powder group (2.8 ± 0.4 min). The PP hemostatic powder was endowed with antioxidant capacity by introducing protocatechuic aldehyde, which can effectively inhibit inflammation and promote wound healing. Therefore, via preparation through a facile lyophilization method, the PP hemostatic powder is expected to find a wide application prospect as a qualified hemostatic powder.

## 1. Introduction

Wound hemostasis is a complex physiological process that relies on multiple factors such as biomolecules, signaling pathways, cell populations cytokines, etc. [1]. In recent years, with the continuous development of wound-management techniques, wound dressings used for hemostasis have attracted worldwide attention [2]. Hydrogels represent three-dimensional networks of hydrophilic polymers cross-linked by chemical and physical bonds that can absorb and retain large amounts of water or biological fluids [3,4]. As soft and moist materials, hydrogels usually have good biocompatibility, strong adhesion, high stretchability, and good self-healing properties [5,6,7,8]. Hydrogel bio-adhesives have a variety of unique advantages, such as good biosafety, degradability, similarity to the natural extracellular matrix, hydrophilicity, moisture retention, flexibility, etc. [9,10,11,12]. Moreover, equipped with diverse functional groups, certain hydrogels can establish covalent or non-covalent bonds with tissue surfaces, becoming one of the most promising candidates for medical wound dressings [13,14,15,16]. In recent years, the hemostatic properties of hydrogels have been extensively studied [17,18,19,20]. For example, Tang and co-workers prepared hemostatic hydrogels based on carboxymethyl chitosan, 2,3,4-trihydroxybenzaldehyde, peptidyl repressing enzyme, and 4-arm poly (ethylene glycol) aldehyde, which showed a promising future for the clinical treatment of full-thickness wounds [21].

Another commonly used hemostatic dressing is hemostatic powder, which has been clinically proven as an ideal engineering material for treating wounds and tissue [22]. Hemostatic powder can not only provide a moist environment for wound healing but also form a protective barrier with blood at the wound interface. The hemostatic powder absorbs large amounts of water and tends to produce repeated hydration, drawing moisture from the tissue into the dressing when in contact with tissue [19]. Meanwhile, the excess water on the surface of the hemostatic powder material can be used as a lubricant to reduce tissue adhesion. In addition, the hemostatic powder is injectable, can be closely adhered to the uneven wound surface, and can effectively prevent wound inflammation [3]. More importantly, the hemostatic powder can bind with anti-bacterial and anti-inflammatory functions to promote the formation of new blood vessels and tissues and help epithelial cells grow to repair wounds [23].

The design of polypeptide-based hydrogel bio-adhesives with intelligent drug-controlled release properties and stimulus responsiveness for chronic wounds is one of the current research hotspots [24]. However, polypeptide-based hydrogel bio-adhesives have limited fluid uptake capacity and are not suitable for highly exudative wounds. Improper application could lead to the excessive accumulation of tissue exudate at the wound site and macerate surrounding healthy tissue, thereby delaying healing and increasing treatment costs. Considering the unique features of hemostatic powder, we designed a composite hemostatic powder based on a mixture of γ-polyglutamic acid (γ-PGA), polyethyleneimine (PEI), N-hydroxysuccinimide (NHS), and 1-(3-dimethylaminopropyl)-3-ethylcarbodiimide (EDC) using a facile lyophilization method. γ-PGA has good hydrophilicity and is able to quickly absorb blood and interstitial fluid when deposited on a bleeding wound and eventually form a stable mechanical hydrogel. Furthermore, the γ-PGA/PEI powder (PP hemostatic powder) was endowed with antioxidant capacity by introducing protocatechuic aldehyde (PCA), which can effectively inhibit inflammation and promote wound healing. This study provides a facile method to prepare multifunctional hemostatic powder, which is anticipated to promote the research and clinical transformation of wound dressing materials.

## 2. Results and Discussion

### 2.1. Preparation and Characterization of the PP Hemostatic Powder

**Figure 1:**
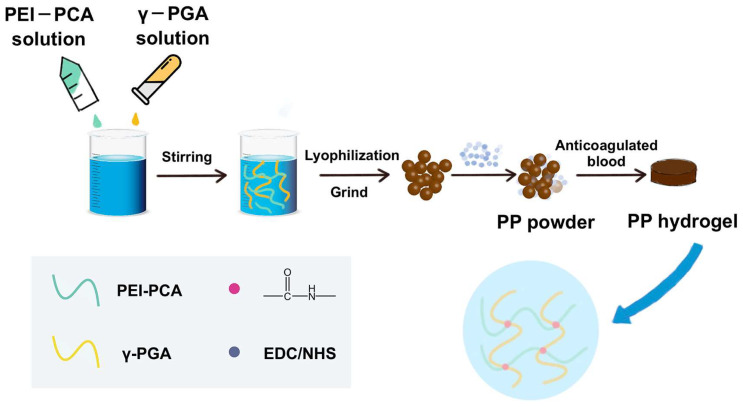
Preparation of the PP hemostatic powder and PP hemostatic powder-derived hydrogel.

The PP hemostatic powder was prepared using a simple lyophilization method. In the powder, γ-PGA has excellent hydrophilicity to quickly absorb blood and then form a hydrogel with PEI under the activation of EDC/NHS (Figure 1). Upon the introduction of PCA, the PP hemostatic powder was endowed with antioxidant capacity, which can effectively inhibit inflammation and promote wound healing.

**Figure 2:**
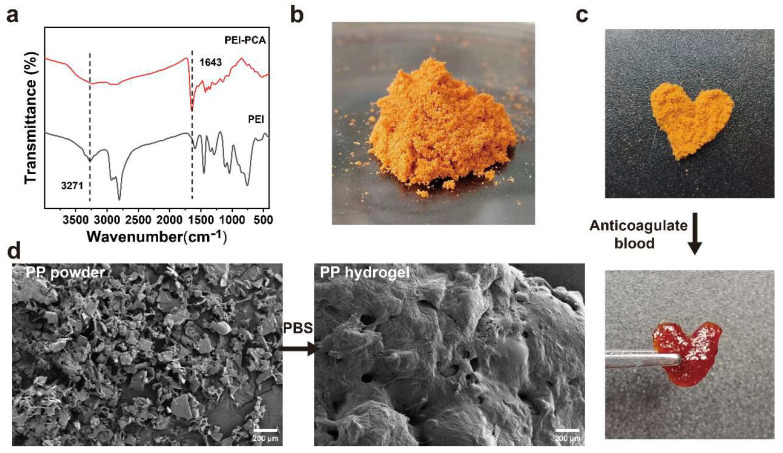
(**a**) Infrared spectra of PEI and PEI-PCA compounds; (**b**) macroscopic image of PP hemostatic powder; (**c**) macroscopic image of PP hemostatic powder absorbing anticoagulant blood to form a hydrogel; (**d**) SEM image of the PP hemostatic powder and PP hemostatic powder-derived hydrogel after absorbing PBS.

The PEI-PCA was analyzed by FTIR. As shown in Figure 2a, comparing the FTIR spectra of PEI and PEI-PCA, it can be seen that PEI-PCA has a new sharp peak at 1643 cm^−l^, and the amino peak in the range of 3100–3500 cm^−1^ becomes weaker, suggesting the formation of Schiff base (C=N) imine bonds. The mixed aqueous solution of PEI-PCA and γ-PGA was lyophilized and mixed with EDC/NHS to prepare the PP hemostatic powder (Figure 2b). The PP hemostatic powder will rapidly expand to form a hydrogel after contact with anticoagulated blood or water. For example, anticoagulant blood was added to heart-shaped PP hemostatic powder, and a heart-shaped PP hydrogel was obtained (Figure 2c). The microstructure of the PP hemostatic powder and PP hemostatic powder-derived hydrogel was studied using SEM. When the PP hemostatic powder became a PP hemostatic powder-derived hydrogel after absorbing water, the hydrogel exhibited a three-dimensional porous structure (Figure 2d). This three-dimensional porous structure can effectively absorb body fluids exuded from the wound, keep the wound area clean and moist, and facilitate the transportation of cell nutrients, thereby promoting hemostasis and wound healing.

### 2.2. Mechanical and Rheological Properties

**Figure 3:**
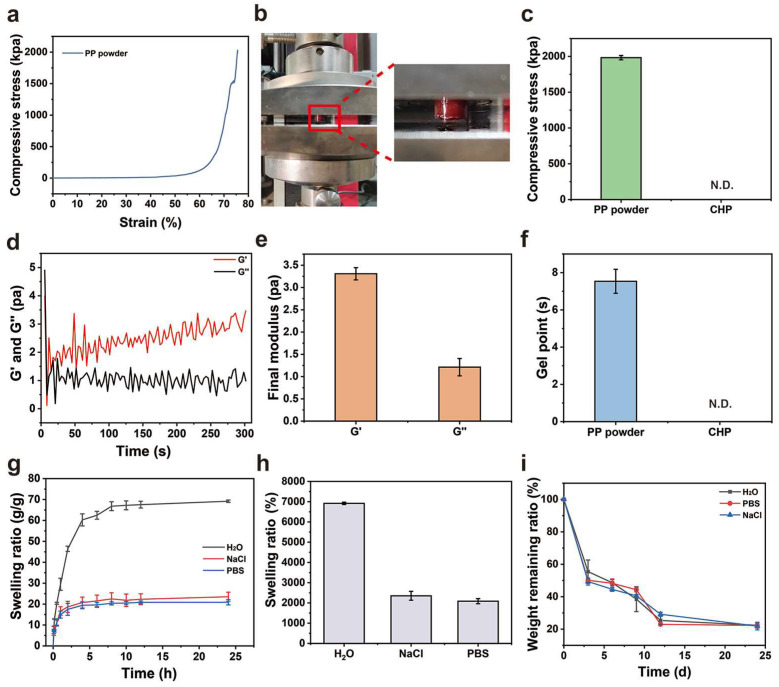
(**a**) Compressive stress–strain curve of the PP hemostatic powder-derived hydrogel; (**b**) schematic diagram of the compression experiment with the PP hemostatic powder-derived hydrogel; (**c**) final compressive strength of the PP hemostatic powder-derived hydrogel and CHP (N.D. = not detectable); (**d**) dynamic change diagram of G′ and G″ after adding PBS to the PP hemostatic powder; (**e**) final G′ and G″ after adding PBS to the PP hemostatic powder; (**f**) gelling time of the PP hemostatic powder and CHP N.D. = not detectable); (**g**) swelling kinetics curve of PP hemostatic powder-derived hydrogel in H_2_O, PBS, and NaCl solutions for 24 h; (**h**) swelling ratio of PP hemostatic powder-derived hydrogel in H_2_O, PBS, and NaCl solutions for 24 h; (**i**) degradation kinetics curve of PP hemostatic powder-derived hydrogel in H_2_O, PBS, and NaCl solutions for 24 h.

As a hemostatic material, the derived hydrogel formed after PP hemostatic powder absorbs body fluids should have strong mechanical properties to serve as a physical barrier to prevent wound bleeding [25,26]. Therefore, we studied the mechanical properties of PP hemostatic powder-derived hydrogels and the gelation time of the PP hemostatic powder. As a hemostatic material, it needs to have the ability to withstand pressure and avoid failure. In order to verify the compression resistance of PP hemostatic powder after absorbing body fluids, compression tests were conducted (Figure 3a,b). As shown in Figure 3c, in the compression experiment, the maximum compressive strength of the PP hemostatic powder-derived hydrogel was 2010.4 ± 20.1 kPa. The gelation time of the PP hemostatic powder was explored through rheological experiments. After adding PBS to the PP hemostatic powder, the G′ curve intersected the G″ curve, and then the G′ was higher than the G″, which shows that a stable hydrogel was formed through cross-linking (Figure 3d). The results showed that the G′ of the PP hemostatic powder-derived hydrogel was about three times that of G″, and the storage modulus and loss modulus of the formed hydrogel were respectively 3.3 ± 0.1 Pa and 1.2 ± 0.2 Pa (Figure 3e). Meanwhile, the rheological curve indicated that the PP hemostatic powder can achieve the transformation from powder to hydrogel within 9 s (Figure 3f). In general, the PP hemostatic powder can quickly absorb water to form a hydrogel with excellent mechanical properties. It is worth mentioning that the CHP forms a high viscosity block, which can quickly adhere to and seal the wound. However, it cannot form a typical hydrogel. Therefore, its mechanical and rheological properties were not detected (Figure 3c,f).

### 2.3. Swelling and Degradation

The good degradation ability of a hemostatic material can avoid secondary damage to the wound [27]. A hemostatic dressing that can effectively absorb tissue fluid and blood will not only increase the concentration of coagulation components at the bleeding site and promote coagulation but also keep the wound moist. Therefore, we performed a swelling experiment to verify the absorption performance of the PP hemostatic powder-derived hydrogel. As shown in Figure 3g,h, the hydrogel reached an equilibrium state after swelling in H_2_O, PBS, and 0.9% NaCl solution for 24 h. The equilibrium swelling ratio in water was the highest, reaching 6918 ± 55.4%. The degree of swelling in PBS and 0.9% NaCl solution was lower than that in H_2_O, which may be due to the presence of salt ions and the higher osmotic pressure of PBS and 0.9% NaCl solution. Meanwhile, the degradation performance of the PP hemostatic powder-derived hydrogel was evaluated. As shown in Figure 3i, the degradation rate of the derived hydrogel was high in the early stage and gradually decreased in the later stage. On day 24, the remaining mass ratios of the derived hydrogel in H_2_O, PBS, and 0.9% NaCl solutions were 22.4 ± 1.7%, 22.2 ± 1.4%, and 21.9 ± 2.4%, respectively, indicating that the PP hemostatic powder-derived hydrogel has good in vitro degradation properties. The degradation can be attributed to the hydrolysis of amide bonds in the hydrogel formed after the water absorption. It is worth mentioning that the CHP cannot form a typical hydrogel. Therefore, its swelling and degradation properties were not studied.

### 2.4. Antioxidant Properties

**Figure 4:**
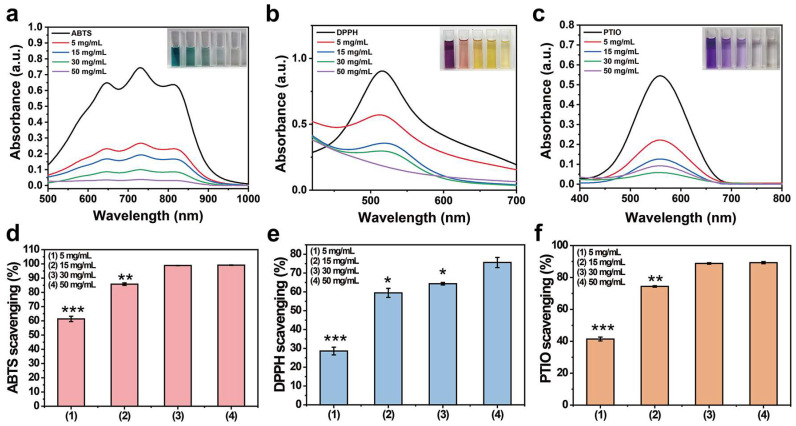
(**a**) UV–visible spectrum of ABTS· after being scavenged by PP hemostatic powder at different concentrations for 3 min; (**b**) UV–visible spectrum of DPPH· after being cleaned by PP hemostatic powder at different concentrations for 30 min; (**c**) PTIO· UV–visible spectrum after being cleaned by PP hemostatic powder at different concentrations for 2 h; (**d**) the clearance rate of ABTS· by PP hemostatic powder at different concentrations; (**e**) the clearance rate of DPPH· by PP hemostatic powder at different concentrations; (**f**) the scavenging rate of PTIO· by PP hemostatic powder at different concentrations. *p* * < 0.05, *p* ** < 0.01, *p* *** < 0.001.

Chronic inflammation at the wound site generates large amounts of reactive oxygen species, which may lead to oxidative stress and prevent angiogenesis and extracellular matrix remodeling [28]. Therefore, a hemostatic material with antioxidant properties will find diverse applications as a wound dressing. The antioxidant properties of PP hemostatic powder were explored through oxygen free radical and nitrogen free radical scavenging experiments. When the PP hemostatic powder concentration reaches 50 mg/mL, the colors of ABTS·, DPPH·, and PTIO· working solutions all fade away and become almost transparent and colorless. The UV–visible spectrum also proves that the characteristic peak absorbance is close to 0 (Figure 4a–c). As the PP hemostatic powder concentration increases, the scavenging rate becomes higher. The maximum scavenging rate for ABTS· is 99.1 ± 0.2% (Figure 4d, *p* < 0.01 versus 15 mg/mL; *p* < 0.001 versus 5 mg/mL), while the maximum scavenging rates for DPPH· and PTIO· are 75.6 ± 2.6% and 89.3 ± 0.3%, respectively (Figure 4e, *p* < 0.05 versus 30 mg/mL, *p* < 0.05 versus 15 mg/mL, *p* < 0.001 versus 5 mg/mL; Figure 4f, *p* < 0.01 versus 15 mg/mL, *p* < 0.001 versus 5 mg/mL). Such antioxidant properties can be attributed to the introduction of PCA with catechol groups. With the proven antioxidant capabilities, the PP power can prevent the excessive expression of free radicals at the wound site, inhibit inflammation, and thereby promote wound healing.

### 2.5. In Vitro Safety

**Figure 5:**
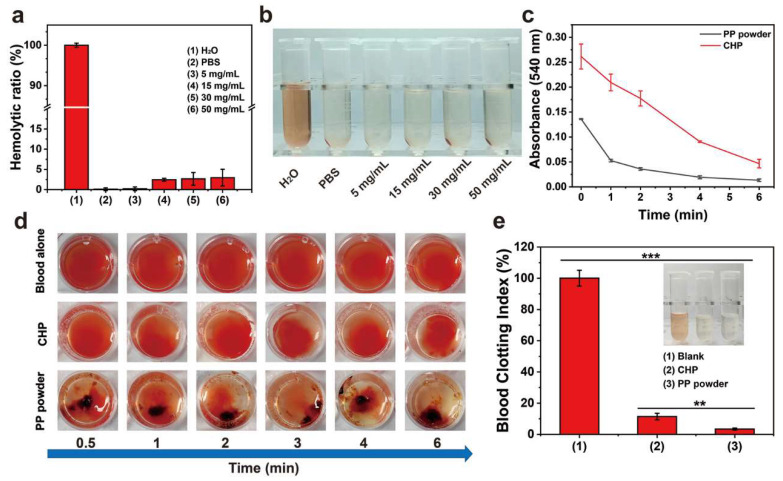
(**a**) Hemolysis ratios of PBS, H_2_O, and PP hemostatic powder at different concentrations; (**b**) photos of centrifuged blood cells treated with PBS, H_2_O, and PP hemostatic powder at different concentrations; (**c**) in vitro whole-blood coagulation kinetics curves of commercial CHP and PP hemostatic powders; (**d**) images of blood coagulation after different treatments; (**e**) blood coagulation index of full blood and blood treated with commercial CHP and PP hemostatic powders. *p* ** < 0.01, *p* *** < 0.001.

An ideal hemostatic material should have no or a small amount of hemolysis when in contact with bleeding wounds. An in vitro hemolysis test was used to evaluate the blood compatibility of the PP hemostatic powder. As shown in Figure 5a,b, after incubation with PP hemostatic powder at 37 °C for 2 h, the supernatants of all centrifuged samples showed a transparency similar to that of the negative control group, without obvious cell disruption. However, the blood treated with H_2_O in the positive control group turned bright red due to hemolysis. The hemolysis ratios of PP hemostatic powders at different concentrations (5, 15, 30, and 50 mg/mL) were all less than 5%, with values of 0.2 ± 0.5%, 2.5 ± 0.3%, 2.7 ± 1.3%, and 3.0 ± 1.6%, respectively, and were considered to represent the safety level of hemostatic materials. These results proved that PP hemostatic powder has good blood compatibility and is a safe hemostatic powder.

### 2.6. In Vitro Hemostasis Experiments

**Figure 6:**
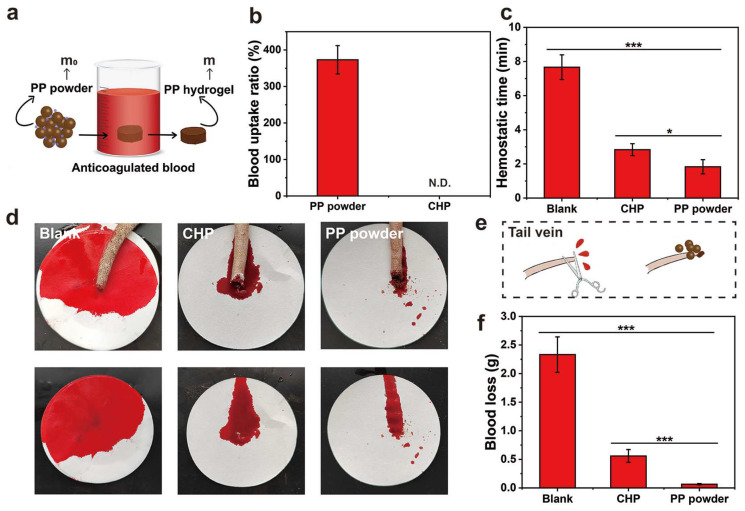
(**a**) Schematic diagram of measuring the blood absorption ratio of the PP hemostatic powder (m and m_0_ are the weight of the PP hemostatic powder and the hemostatic powder-derived hydrogel); (**b**) blood uptake ratio of PP hemostatic powder and CHP (N.D. = not detected); (**c**) tail-vein bleeding time of SD rats in the blank group, CHP group, and PP hemostatic powder group; (**d**) corresponding to (**c**) photo of tail-vein hemostasis; (**e**) schematic diagram of tail-vein hemostasis with PP hemostatic powder; (**f**) tail-vein blood loss of SD rats in the blank group, CHP group, and PP hemostatic powder group. *p* * < 0.05, *p* *** < 0.001.

In order to explore the coagulation ability of PP hemostatic powder, an in vitro whole-blood coagulation experiment was conducted. The whole-blood coagulation kinetics of PP hemostatic powder and commercial CHP powder were evaluated by measuring the absorbance of the supernatant of PP hemostatic powder- and commercial CHP powder-treated blood. As shown in Figure 5c, the PP hemostatic powder group adsorbed some red blood cells initially, and the absorbance value was much lower than that of the CHP group. The absorbance of the PP hemostatic powder group decreased significantly at 1 min until the absorbance was close to 0 at 6 min. The absorbance value representing the hemoglobin concentration decreased as the blood began to coagulate, and the rapid decrease corresponded to a high coagulation rate. Figure 5d records the blood coagulation conditions of PP hemostatic powder, CHP, and blank groups at different time points (0.5, 1, 2, 3, 4, and 6 min). It can be seen that the blood in the blank group and CHP group was diffused red in the water, while the supernatant in the PP group remained transparent after 1 min. Blood coagulation is a dynamic process in which coagulation factors are effectively activated and ultimately convert fibrinogen into fibrin. During the coagulation process, red blood cells are captured by the irregular pores of the PP hemostatic powder, and a large amount of fibrin aggregates to form a fibrin matrix, which captures more blood cells [29,30]. Finally, the blood clot formed by PP hemostatic powder showed a dark red color composed of blood cells and fibrin. As shown in Figure 5e, both the blank group and the CHP group showed higher blood coagulation indices, indicating poor hemostatic performance. The blood coagulation index value of the PP hemostatic powder group was significantly lower than those of the blank group and CHP group, only 3.4 ± 0.6% (*** *p* < 0.01, versus CHP; *** *p* < 0.001, versus blank), indicating a better in vitro hemostatic performance. To further explore the blood absorption ability of the PP hemostatic powder, a blood absorption experiment was conducted. The experimental operation is shown in Figure 6a. After 30 s, the blood absorption rate of the PP hemostatic powder was as high as 373.1 ± 38.8%, which shows that it has strong blood absorption ability and is a potential hemostatic material. Because the CHP cannot form a typical hydrogel, the blood uptake ratio of CHP was not studied (Figure 6b).

### 2.7. In Vivo Hemostasis Experiments

The in vivo hemostatic properties of the PP hemostatic powder were evaluated through the rat tail-docking model. After treatments, the bleeding time of the PP hemostatic powder group was 1.8 ± 0.4 min (* *p* < 0.05, versus CHP; *** *p* < 0.001, versus blank), while the bleeding time of the CHP group was 2.8 ± 0.4 min. However, the untreated blank control group needed 7.7 ± 0.7 min to stop bleeding (Figure 6c). The schematic diagram of the tail-docking experiment can be seen in Figure 6e. As shown in Figure 6d,f, without any hemostatic treatment, the blood loss of tail-docked rats was 2.3 ± 0.3 g within 10 min. Although CHP can absorb blood, it cannot form a stable physical barrier and dissolve in the blood, resulting in a blood loss of 0.6 ± 0.1 g. The PP hemostatic powder can absorb a large amount of blood, concentrate coagulation factors, gather blood cells and platelets, and achieve the lowest blood loss. Therefore, the blood loss in wounds treated with the PP hemostatic powder was only 0.06 ± 0.01 g (*** *p* < 0.001, versus CHP; *** *p* < 0.001, versus blank). These results indicate that the PP hemostatic powder has good in vivo hemostatic ability and is suitable for complex bleeding sites with irregular geometries.

## 3. Conclusions

In summary, we successfully synthesized a multifunctional PP hemostatic powder that can resist inflammation and hemostasis, which is expected to contribute to wound healing. When the PP hemostatic powder is deposited on a bleeding wound, it can quickly absorb a large amount of blood and interstitial fluid, concentrate coagulation factors, coagulate blood cells, and quickly form (<9 s) a stable and strong mechanical hydrogel (maximum compressive strength of 2010.4 ± 20.1 kPa). In addition, due to the presence of PCA, PP hemostatic powder is endowed with antioxidant capacity, which can effectively inhibit inflammation and promote wound healing. PP hemostatic powder also showed good coagulation and hemostasis performance in vitro. The rat-tail venous hemorrhage model showed that its hemostatic performance was better than that of the commercial product CHP. Therefore, we believe that PP hemostatic powder is expected to become a new hemostatic material and wound dressing and has a wide application prospect.

## 4. Materials and Methods

### 4.1. Materials

PEI (Mw: 70 kDA) aqueous solution (30 wt.%) was purchased from Thermo Fisher (Shanghai, China). PCA (≥98%) was purchased from Shanghai Yuanye Biotechnology Co., Ltd. (Shanghai, China). γ-PGA (Mw: 700 kDa) was purchased from Shanghai Yika Biotechnology Co., Ltd. (Shanghai, China). Calcium (CaCl_2_), EDC, NHS, the (2,2′-azino-bis(3-ethylbenzothiazoline-6-sulfonic acid radical (ABTS·), 1,1-diphenyl-2-picrylhydrazyl free radicals (DPPH·), and 2-phenyl-4,4,5,5-tetramethylimidazoline-1-oxyl 3-oxide (PTIO·) were purchased from Shanghai Aladdin Reagent Co., Ltd. (Shanghai, China). Commercial chitosan hemostatic powder (CHP) was bought from Qingdao Biotemed Biomaterials Co., Ltd.

### 4.2. Preparation of the PP Hemostatic Powder

A 1 mL volume of PEI was diluted in 20 mL of deionized water and stirred evenly at room temperature. Then, 0.075 g of PCA was added to the above solution and stirred at room temperature for 4 h to obtain a solution. The reaction solution was dialyzed in deionized water for 3 days and lyophilized to obtain PEI-PCA powder. To prepare the PP hemostatic powder, PEI-PCA (1 wt.%, 4 mL, in distilled water) and γ-PGA (1 wt.%, 4 mL, in distilled water) solutions were mixed, and 16 mL of deionized water was added thereinto. Then, the mixed solution was placed in a freeze dryer for 72 h for lyophilization. Finally, 0.04 g EDC and 0.04 g NHS were added thereinto and grinded using a grinder for 5 min to obtain PP hemostatic powder. The PP hemostatic powder was stored in a 4 °C refrigerator for later use.

### 4.3. Characterization

The chemical structure of PEI-PCA was first analyzed by Fourier transform infrared spectroscopy (FTIR). Then, PP hemostatic powder was mixed with PBS to prepare PP hydrogel (PP hemostatic powder-derived hydrogel). After lyophilization, the morphology of the PP hemostatic powder was observed under a scanning electron microscope (SEM). In addition, a hydrogel formed from the PP hemostatic powder was recorded during the full absorption of mouse blood.

### 4.4. Mechanical Testing

In order to verify the compressive resistance of the hydrogel formed from the PP hemostatic powder, a general material testing machine (Zwick Roell Z2.5 TH with 2.5 kN sensor) for compressive stress–strain measurement was used. In the experiment, the PP hydrogel was prepared in a cylindrical shape with a diameter of 10 mm and a depth of 4 mm. At a predetermined compressive strain rate of 1 mm/min, the hydrogel was compressed to 80% of the initial height to measure its compressive properties, with a strain range of 0 to 90%. All these tests were performed three times in parallel.

In order to study the hydrogel formation process with the PP hemostatic powder, a parallel plate (P20 TiL, diameter 20 mm) rotational rheometer (MARS III HAAKE) was used to conduct dynamic rheological studies. Briefly, the PP hemostatic powder was placed on parallel plates preheated to 37 °C. Subsequently, PBS was added dropwise to the powder. After the powder and PBS were evenly mixed, a time-sweep test was performed at 37 °C (simulated body temperature) with a constant frequency of 1 HZ and a strain of 1%. The storage modulus G′ and loss modulus G″ were analyzed. Meanwhile, when the G′ curve exceeded the G″ curve, the gelation time point was determined.

### 4.5. Study of Swelling Behaviors

In this section, a swelling experiment was used to measure the swelling rate of the PP hemostatic powder-derived hydrogel. The freeze-dried hydrogel blocks (0.3 g) were placed into 25 mL H_2_O, PBS, and 0.9% NaCl solution (*n* = 3), and incubated in a 37 °C constant-temperature incubator. At predetermined time intervals, the hydrogel was removed from the solution, and excess water on the surface of the hydrogel was absorbed with filter paper and then weighed to plot the swelling kinetics curve. The swelling ratio was calculated when the hydrogel reached swelling equilibrium using the calculation formula:

$$\mathrm{Swelling}\;\mathrm{ratio}\;\left(\%\right)=\frac{W_{t}-W_{0}}{W_{0}}\times 100\%$$

where *W*_0_ and *W_t_* are the initial weight of the hydrogel and the weight after swelling at different time points, respectively.

### 4.6. In Vitro Degradation

In vitro degradation experiments were conducted to verify the biodegradability of the PP hemostatic powder. The prepared PP hemostatic powder-derived hydrogel was freeze dried and incubated with 10 mL of H_2_O, PBS, and 0.9% NaCl solution containing lysozyme (10^4^ U/mL, *n* = 3) in a constant-temperature shaker for 24 days (37 °C, 120 rpm). All solutions were replaced with fresh solutions containing lysozyme every 2 days. On days 3, 6, 9, 12, and 24, hydrogel samples were taken out, the surface was washed with deionized water, and then the sample were lyophilized and weighed. The degradation efficiency of the PP hemostatic powder-derived hydrogel was calculated and expressed as weight remaining (%). The calculation formula is:

$$\mathrm{Weight}\;\mathrm{remaining}\;\left(\%\right)=\frac{W_{t}}{W_{0}}\times 100\%$$

where *W*_0_ and *W_t_* are the initial weight of the lyophilized hydrogel and the dry weight of the remaining hydrogel after degradation at different time points, respectively.

### 4.7. Antioxidant Properties

In order to evaluate the antioxidant properties of PP hemostatic powder, ABTS-, DPPH·-, and PTIO·-scavenging experiments with PP hemostatic powder-derived hydrogels at different concentrations were conducted [31,32].

#### 4.7.1. ABTS· Scavenging Experiment

The overall antioxidant capacity of the PP hemostatic powder-derived hydrogel was determined through an ABTS· free radical scavenging experiment. Firstly, the ABTS· working solution was prepared and diluted with PBS to the absorbance of 0.70 ± 0.02 (wavelength = 734 nm) [33]. PP hemostatic powder-derived hydrogels (weight: 15, 45, 90, and 150 mg) were placed into 3 mL of diluted ABTS· working solution and let stand in the dark for 3 min at room temperature. The sample without hydrogel was used as a blank. Using a UV–visible near-infrared spectrometer, the absorbance of the mixture from 500 to 1000 nm was measured. In addition, a camera was used to record macroscopic images of the color changes in the mixed solution during the experiment. The ABTS·-scavenging efficiency was calculated as follows:

$$\mathrm{ABTS}\cdot \mathrm{scavenging}\;\left(\%\right)=\frac{A_{0}-A_{t}}{A_{0}}\times 100\%$$

where *A_t_* and *A*_0_ are the absorbance of the experimental group and the absorbance of the blank group, respectively.

#### 4.7.2. DPPH· Scavenging Experiment

The ability of the PP hemostatic powder-derived hydrogel to scavenge nitrogen free radicals was determined using a DPPH· scavenging experiment. The DPPH· working solution was prepared and diluted with ethanol solution to an absorbance value of 0.6–1.0 at 517 nm [34]. Then, PP hemostatic powder-derived hydrogels with different concentrations (weight: 15, 45, 90, and 150 mg) were added to 3 mL of diluted DPPH· working solution and let stand in the dark at room temperature for 30 min. The sample without hydrogel was used as a blank. We then used a UV–visible near-infrared spectrometer to measure the absorbance of the mixture and scan the wavelength information of the mixture from 400 to 700 nm. Meanwhile, a camera was used to record macroscopic images of the color changes in the mixed solution during the experiment. The calculation formula for DPPH·-scavenging efficiency is as follows:

$$\mathrm{DPPH}\cdot \mathrm{scavenging}\;\left(\%\right)=\frac{A_{0}-A_{t}}{A_{0}}\times 100\%$$

where *A_t_* and *A*_0_ are the absorbance of the experimental group and the absorbance of the blank group, respectively.

#### 4.7.3. PTIO· Scavenging Experiment

The oxygen free radical-scavenging ability was studied via the PTIO· scavenging method. Firstly, the PTIO· working solution was diluted with deionized water to the absorbance of 0.2–0.6 at 557 nm [35]. Then, PP hemostatic powder-derived hydrogel with different concentrations (weight: 15, 45, 90, and 150 mg) were added to 3 mL of diluted PTIO· working solution and placed in a 37 °C constant-temperature incubator for 2 h. The sample without hydrogel was used as a blank. We used a UV–visible near-infrared spectrometer to measure the absorbance of the mixture from 400 to 800 nm. At the same time, a camera was used to record macroscopic images of the color changes in the mixed solution during the experiment. The calculation formula for PTIO·-scavenging efficiency is as follows:

$$\mathrm{PTIO}\cdot \mathrm{scavenging}\;\left(\%\right)=\frac{A_{0}-A_{t}}{A_{0}}\times 100\%$$

where *A_t_* and *A*_0_ are the absorbance of the experimental group and the absorbance of the blank group respectively.

### 4.8. Blood Compatibility

In order to verify the biocompatibility of PP hemostatic powder, blood compatibility experiments were conducted using Kunming mouse blood. Mouse blood was centrifuged (3000 rpm, 5 min) and washed three times with PBS to collect mouse red blood cells. We diluted the obtained red blood cells to 2% with PBS and stored them in the refrigerator for later use. Then, we added 5, 15, 30, and 50 mg PP hemostatic powder-derived hydrogels to the mixed solution of 2.4 mL of PBS and 0.6 mL of red blood cells, respectively. Another two groups were defined as the control group, that is, the red blood cell suspension was treated with PBS (negative control) and H_2_O (positive control). Then, we transferred all the above samples to a 37 °C constant-temperature incubator and cultured them for 2 h. Finally, all samples were centrifuged (3000 rpm, 5 min) to extract the supernatant. Using a UV–visible near-infrared spectrometer to detect the absorbance of the supernatant at a wavelength of 541 nm, we were then able to calculate the hemolysis ratio according to the formula:

$$\mathrm{Hemolytic}\;\mathrm{ratio}\left(\%\right)=\frac{A_{x}-A_{n}}{A_{t}-A_{n}}\times 100\%$$

where *A_x_* is the absorbance of the supernatant co-cultured with PP hemostatic powder, *A_n_* is the absorbance of the negative control, and *A_t_* is the absorbance of the positive control.

### 4.9. In Vitro Hemostasis Test

A 20 µL volume of anticoagulated mouse whole blood was dropped into 10 mg of PP hemostatic powder, and then 1 µL of CaCl_2_ solution (0.1 mol/mL) was added to initiate blood coagulation. Similarly, the above-mixed blood was added to CHP in the control group. In this experiment, no materials were added to the blank group, and the mixed blood coagulated naturally. The three groups of materials were transferred to a 37 °C constant-temperature incubator and taken out at preset time points (0.5, 1, 3, 4, and 6 min). By slowly adding deionized water (5 mL) to rinse the samples to allow dissociation, the red blood cells were broken up and pictures were taken. The absorbance of the supernatant at a wavelength of 541 nm was detected by a UV–visible near-infrared spectrometer, and the whole-blood coagulation kinetics curve was drawn. The blood coagulation index was calculated according to the formula:

$$\mathrm{Blood}\;\mathrm{clotting}\;\mathrm{index}\;\left(\%\right)=\frac{A_{s}}{A_{0}}\times 100\%$$

where *A_s_* is the absorbance of the supernatant of the material group, and *A*_0_ is the absorbance of the supernatant of the blank group.

### 4.10. In Vivo Hemostasis Study

To study blood absorption, PP hemostatic powder with weight m_0_ was added to 2 mL of anticoagulated whole blood at 37 °C for 30 s [36,37]. Then, the PP hemostatic powder-derived hydrogel was taken out, and excess Kuming mouse blood was wiped off with filter paper. The weight was recorded as m. The blood absorption ratio was calculated as follows (*m* and *m*_0_ are the weight of the PP hemostatic powder and the hemostatic powder-derived hydrogel, respectively):

$$\mathrm{Blood}\;\mathrm{uptake}\;\mathrm{ratio}\;\left(\%\right)=\frac{m}{m_{0}}\times 100\%$$

An SD rat tail-docking model was used to evaluate the hemostatic potential of PP hemostatic powder. All experimental protocols and animal handling were conducted following the regulations of the Animal Investigation Ethics Committee of Changhai Hospital. Rats were randomly divided into 3 groups (*n* = 3) and anesthetized with an intraperitoneal injection of chloral hydrate (4%). The experimental group used PP hemostatic powder for hemostasis, the control group used CHP for hemostasis, and the blank group did not undergo any hemostasis treatment. A weighed filter paper was placed at the bleeding site 8 cm from the tail of the rat to absorb blood. After 3 min, photos of the wound were recorded. Finally, the filter paper was weighed again to calculate the blood loss. The hemostasis times of the blank group, control group, and PP hemostatic powder group were measured respectively. All experiments were repeated three times.

### 4.11. Statistical Analysis

All data are expressed as mean ± standard deviation of at least triplicate samples. Differences between independent groups were analyzed using multiple *t*-tests (*p* * < 0.05, *p* ** < 0.01, *p* *** < 0.001).

## Data Availability

The data presented in this study are openly available in article.
